# High-fat-diet impaired mitochondrial function of cumulus cells but improved the efficiency of parthenogenetic embryonic quality in mice

**DOI:** 10.1080/19768354.2018.1497707

**Published:** 2018-08-22

**Authors:** Jingjing Li, Shuang Wang, Bo Wang, Hao Wei, Xin Liu, Jun Hao, Yanping Duan, Jinlian Hua, Xiaomin Zheng, Xiuliang Feng, Xingrong Yan

**Affiliations:** a College of Life Sciences, Northwest University, Xi’an, People’s Republic of China; b Department of Experimental Surgery of Xijing Hospital, Fourth Military Medical University, Xi’an, People’s Republic of China; c Biotechnology, Northwest A&F University, Yangling, People’s Republic of China; d Key Laboratory of Fertility Preservation and Maintenance, Ministry of Education, Key Laboratory of Reproduction and Genetics in Ningxia, Ningxia Medical University, Yinchuan, People’s Republic of China; e Department of Histology and Embryology, Ningxia Medical University, Yinchuan, People’s Republic of China

**Keywords:** Embryo, high-fat diet, mouse, oocyte, parthenogenesis

## Abstract

Global human health has been compromised by high-fat diets. This study aimed to investigate the relationship between a high-fat diet and parthenogenetic embryo quality. Mice fed a high-fat or a normal diet was used as treated or control groups, respectively. Estradiol (E_2_), total cholesterol (TC) and total triglyceride (TG) were detected by Enzyme-Linked ImmunoSorbent Assay (ELISA). Cumulus-oocyte complexes (COCs) were collected from the mice in the treated and control groups. The ultrastructure of COCs, the expression level of genes involved in mitochondrial and nuclear functions in cumulus cells and oocytes quality were evaluated with transmission electron microscopy, real-time quantitative polymerase chain reaction (RT-PCR) and artificial parthenogenesis, respectively. The results showed that the efficiency of parthenogenetic embryonic development in vitro was significantly higher in the treated group than in the control group (*p* < .05). The expression level of genes involved in mitochondrial function was lower in cumulus cells from the treated group than that from the control group (*p* < .05). The estradiol and cholesterol level in the serum and the expression level of *P450 arom* were higher in the treated group than the control group (*p* < .05). The reactive oxygen species (ROS) level was higher in culumus cells from the treated group than the control group, while the mitochondrial membrane potential was lower in cumulus cells from the treated group (*p* < .05). Accumulation of lipid droplets was only in cumulus but in oocyte, the results demonstrated that mitochondrial functions were impaired by a high-fat diet, but parthenogenetic embryonic development in vitro was improved, in controllable range of damage for the body.

## Introduction

Obesity is an increasing public health concern for humans, as it can cause many diseases, such as diabetes, cardiovascular disease and other metabolic diseases at rapid rates (Kulie et al. 2011). Since it is directly related to the health of the next generation, women’s health is gradually becoming more of a public focus compared with men’s health. Therefore, obesity is an important cause of many diseases in women (Thangaratinam et al. 2012). More importantly, obese women have high rates of infertility compared with their lean counterparts. However, obesity could be caused by many factors, such as consumption of excess calories and calorie-dense foods, sedentary behavior and intake of a high-fat diet (Rey-López et al. 2008). In obesity, lipids could assemble in non-adipose tissues, such as skeletal muscle, liver, and heart, due to increased cellular uptake of exogenous fatty acids (Cuevas et al. 2004). Increased levels of intracellular free fatty acids in the absence of obesity could also lead to the formation of cytotoxins due to oxidative damage, especially for intracellular organelles, such as mitochondria and the endoplasmic reticulum (ER) (Borengasser et al. 2011). One of these organelles, the mitochondria, is the intracellular energy-generating organelle that determines cell activity. Therefore, mitochondria are important for the development of oocytes and embryos. Obesity can also lead to insulin resistance and inflammatory changes (Shoelson et al. 2007). Insulin resistance increases the level of glucose in the body, and moreover, lipogenesis could be enhanced to protect against high intracellular glucose levels. Inflammatory changes could be triggered by obesity, as adipocyte can secrete pro-inflammatory cytokines, such as tumor necrosis factor α (TNFα) and monocyte chemoattractant protein-1 (MCP-1), which are relevant to insulin resistance (Kim et al. 2012). Thus, obesity is a vicious cycle in animals and is difficult to control through a single method.

Extensive investigations have focused on the effect of obesity on the reprogramming of embryos during pregnancy (Srinivasan et al. 2008). The status of embryo development at the early stage, especially preimplantation, is an index used to evaluate embryo developmental competence. Early embryonic development is supported and controlled by the oocyte, which is specifically a very large germ cell. An oocyte maintains its normal activities with help from the cumulus cells surrounding it. Therefore, oocytes may be directly affected by cumulus cells, as these cells are linked by gap junctions between them (Lisle et al. 2013). The nutrition and hormones of the oocyte are supported by cumulus; for example, cumulus cells can secrete E_2_ via aromatizing enzyme. Many attempts have been made to predict oocyte quality by identifying cumulus cell states (Wathlet et al. 2012). Previous reports showed that high insulin could promote glucose uptake in cumulus cells, rather than oocytes. Cumulus cells control the metabolism of glucose and provide energy substrates and intermediates, such as pyruvate, to the oocyte (Purcell et al. 2012). Sufficient energy can maintain an oocyte and better support for embryo development. Previous reports have used adult animal models to investigate the relationship between a high-fat diet and reproduction (Wu et al. 2010). In this study, 4-week-old mice were fed a high-fat diet to determine whether these results are the same as those in adult animal models.

## Materials and methods

### Animals

Female ICR mice (4 weeks old) were purchased after ablactation. The mice were divided into treated group and control group with 30 mice respectively. Mice were raised under the following conditions: light/dark: 12 h/12 h. All handling procedures were carried out in accordance with the guidelines of the Experimental Animal Holding Unit of fourth Military Medical University (ethics approval number 2006731019). A high-fat diet was prepared as described in a previous report; briefly, the diet contained 22% fat, 19% protein, and 49.5% carbohydrate (Wu et al. 2010). Female mice in a treated group were prepared for use after being fed a high-fat diet for 4 weeks, while the the mice in control group were fed the normal food with 4.5% fat, 19% protein and 5% carbohydrate as a control.

### Histochemical stains and ultra structural analysis of COCs

COCs were collected from the oviducts of treated and control groups 16 h after super-ovulation. And then the ovaries and cornua uteri were harvested from mice after collection of COCs. Ovaries and cornua uteri were fixed with 4% paraformaldehyde for 48 h, dehydrated with different concentrations of ethanol, and embedded in paraffin. The paraffin-embedded tissues were cut into 5-µm slices with a Leica RM 2165. Subsequently, the sections were deparaffinized, rehydrated and stained with hematoxylin–eosin (Lee et al. 1999). The procedure was performed by the improved method from the previous report. The COCs were fixed for 2 d in phosphate buffered solution (PBS) containing 2.5% glutaraldehyde, then fixed for 2 h in 1% osmium tetroxide in PBS (Palmerini et al. 2014). The specimens were dehydrated in an increasing series of ethanol, infiltrated with a propylene oxide, and embedded in Epon-Araldite (Epon-812) (TAAB, UK). Ultrathin sections (70 nm) were cut with a diamond knife, stained with 3% uranyl acetate and 0.6% lead citrate, and examined with a transmission electron microscope (Chung and M⁠oon 2011).

### Detection of e_2_, cholesterol and triglyceride (TG) levels in serum

Detection of E_2_, TC and TG was performed as previously described (Schliep et al. 2015). Blood was collected from the heart when COCs were recovered from the mice. The serum was separated from the blood by centrifugation and was preserved at –20°C for further use. The level of E_2_, TC and TG were determined using an ELISA Kit (Joyee Biotechnics, Shanghai, China), and the absorbance was detected with a microplate reader, according to the manufacturer’s instructions (infinite F50, Tecan, Switzerland).

### Artificial parthenogenetic activation and cultivation of oocytes

Oocytes were artificially activated by culturing in Ca^2+^-free Chatot & Ziomek & Bavister (CZB) medium with 10 mM SrCl_2_ and 5 µg/ml cytochalasin B for 6 h (Yan et al. 2013). The activated oocytes were cultured in potassium simplex optimization medium (KSOM) over-laid with mineral oil in an incubator (37°C, 5% CO_2_ and saturated humidity). The quality of parthenogenetic embryos was assessed using morphological criteria at 3.5 d.

### Extraction of mRNA and quantitative polymerase chain reaction (qPCR)

Extraction of total RNA from cumulus cells and qPCR were performed as described in a previous report (Jin et al. 2015). Briefly, primers were designed from the published sequence in GenBank. RNA quality was determined using the *β-actin* gene, which was amplified with specific primers (across introns). The primer sequences are shown in Table 1. RT-PCR was performed using a Light Cycler (Roche Molecular Biochemicals, Mannheim, Germany) and a commercially available SYBR-Premix Ex TaqTM II kit (Takara, Japan) according to the manufacturer’s instructions. After the addition of reagents (final volume: 50 μL), 40 cycles of denaturation (94°C for 1 s), annealing (59°C for 10 s), and extension (72°C for 10 s) were performed. After the completion of PCR amplification, melt curve analysis was performed. The experiment was repeated for three times.

**Table 1.** Oligonucleotide primer sets used for RT-PCR

Gene	Primer	Primer sequence	Product
*bax*	Forward primer	5′-TACAGGGTTTCATCCAGG-3′	167bp
* *	Reverse primer	5′-GTCAGCAATCATCCTCTG-3′	
*bcl2*	Forward primer	5′-GAGTTAGTTCGTCTGAGTAG-3′	112bp
* *	Reverse primer	5′-ATAGGTCAAGAGGGAGTG-3′	
*Sox2*	Forward primer	5′-TACAGGGTTTCATCCAGG-3′	167bp
* *	Reverse primer	5′-GTCAGCAATCATCCTCTG-3′	
*P450arom*	Forward primer	5′ ATC AAG CAG CAT TTG GAC CG 3′	140bp
* *	Reverse primer	5′ ACA ATA GCA CTT TCG TCC AG 3′	
*C-myc*	Forward primer	5′-ACTTCTCCACCGCCGATCAG-3′	211bp
* *	Reverse primer	5′-AGGCTGGTGCTGTCTTTGCG-3′	
*Tim23*	Forward primer	5′ CTGACT GGTATGAACCCCCT 3′	121bp
* *	Reverse primer	5′ CTAGTTCAAATCTGCCTCGG 3′5	
*Pnpt1*	Forward primer	5′ CCCACAAACTACCTTAGAAG 3′	191bp
* *	Reverse primer	5″- GCTACAGAAGCACCATTAAC-3′	
*Cyto C*	Forward primer	5′ TTCAGAAGTGTGCCCAGTGC 3′	147bp
* *	Reverse primer	5′ TCCCCCCGTTACCTTTGTTC 3′	
*Cox II*	Forward primer	5′ CCTCTCTACGCATTCTATAT 3′	124bp
* *	Reverse primer	5′ GAATCA AAG CATAGGTCT TC 3′	
*β-actin*	Forward primer	5′-GCGGCATCCACGAAACTAC-3′	120bp
* *	Reverse primer	5′-TGATCTCCTTCTGCATCCTGTC-3′	
*GAPDH*	Forward primer	5′ AGAAGGTGGTGAAGCAGGCA 3′	111bp
* *	Reverse primer	5′ CGAAGGTGGAAGAGTGGGAG 3′	

### Identification of reactive oxygen species (ROS) and mitochondrial membrane potential in cumulus cells

For ROS, COCs were collected from the oviduct, washed in M2 medium for two times, cultured in 2,7-Dichlorodi–hydrofluorescein diacetate (DCFH-DA) medium for 30 min in an incubator (37°C, 5% CO_2_ and saturated humidity), and washed in M2 medium for three times to eliminate DCFH-DA. COCs were observed using a confocal microscope and analyzed by IPP. For mitochondrial membrane potential, COCs were treated with the following methods. COCs were evaluated with a mitochondrial membrane potential kit, which was used according to manufacturer’s instructions in their manual. Briefly, COCs were cultured in 5,5′,6,6′-Tetrachloro-1,1′,3,3′-tetraethyl-imidacarbocyanine iodide (JC-1) work buffer for 20 min (37°C, 5% CO2 and saturated humidity) and washed in JC-1 wash buffer (without JC-1) for three times. COCs were observed under a confocal microscope, and the fluorescence was quantified by IPP software (Media Cybernetics, Bethesda, MD, USA).

## Results

### Structure of the ovary and ultrastructure of cumulus cells

**Figure 1.**
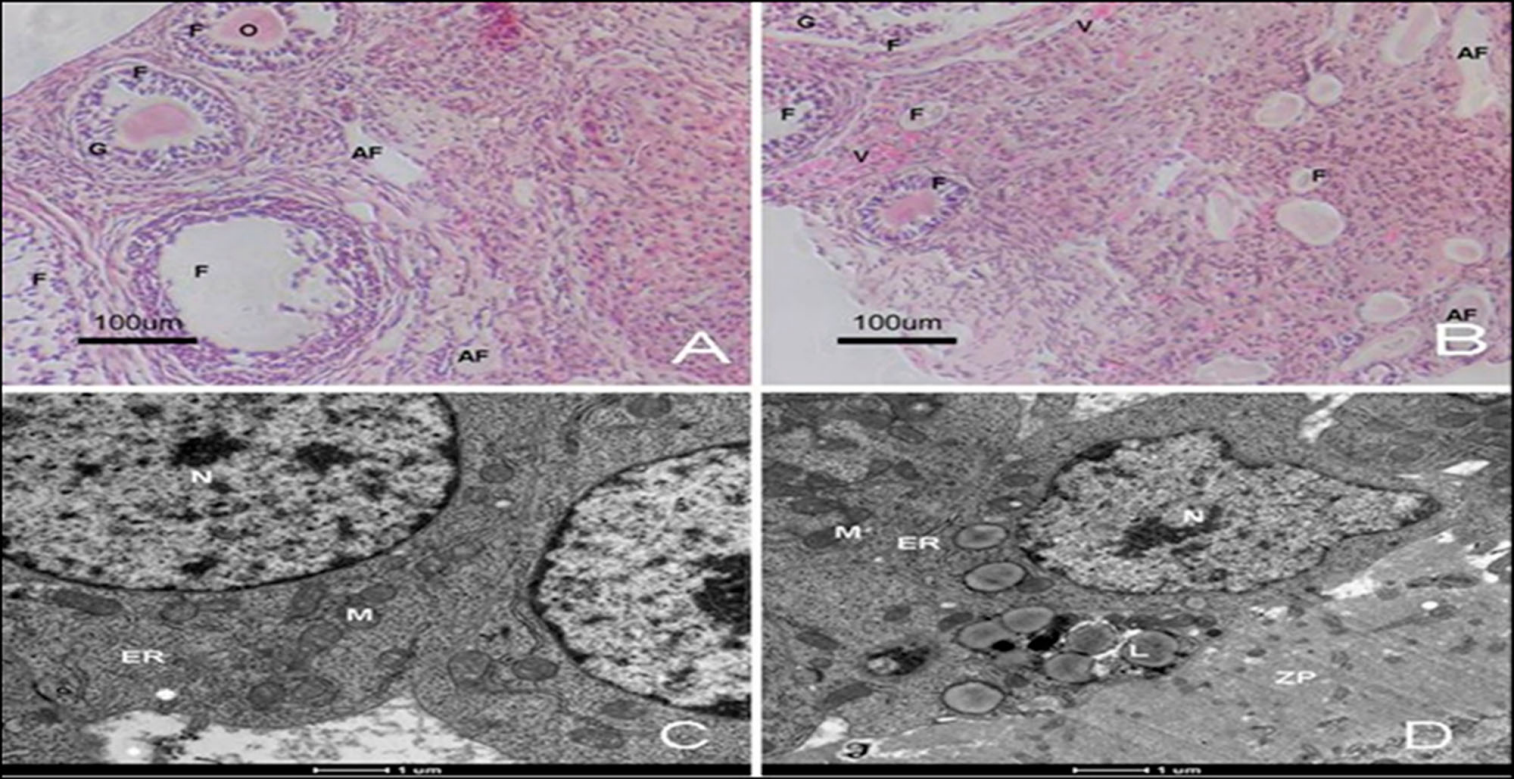
**Structure of ovary and ultrastructure of cumulus in treated and control mice.** HE staining showed that different stages of follicles were present in ovaries from treated (A) and control (B) mice. The ultrastructure of cumulus cells was observed by SEM (C and D); many diploids were assembled in cumulus cells from treated mice (D). F: follicle, G: granule, O: oocyte, V; vasculature, AF: atretic follicle, L: lipid droplet, N: nucleus, M: mitochondrion, ER: endoplasm reticulum.

In the ovary, there were more follicles in the treated group than the control group, and more vasculature was observed in the ovaries from the treated group (Figure 1(A,B)). To analyze the effects of a high-fat diet on the nucleus and cytoplasm of cumulus cells in COCs, the observation was noted: the nucleus was located at the center of cells, and the nuclear membrane was intact in both groups. Mitochondrial, the ER and ribosomes were distributed in the cytoplasm. All the mitochondria had clear cristae. In the treated group, there were several large lipid droplets and more ribosomes in the cytoplasm of cumulus cells (Figure 1(C,D)).

### E_2_, cholesterol and TG level in the serum and expression of *p450 arom* in cumulus cells

**Figure 2.**
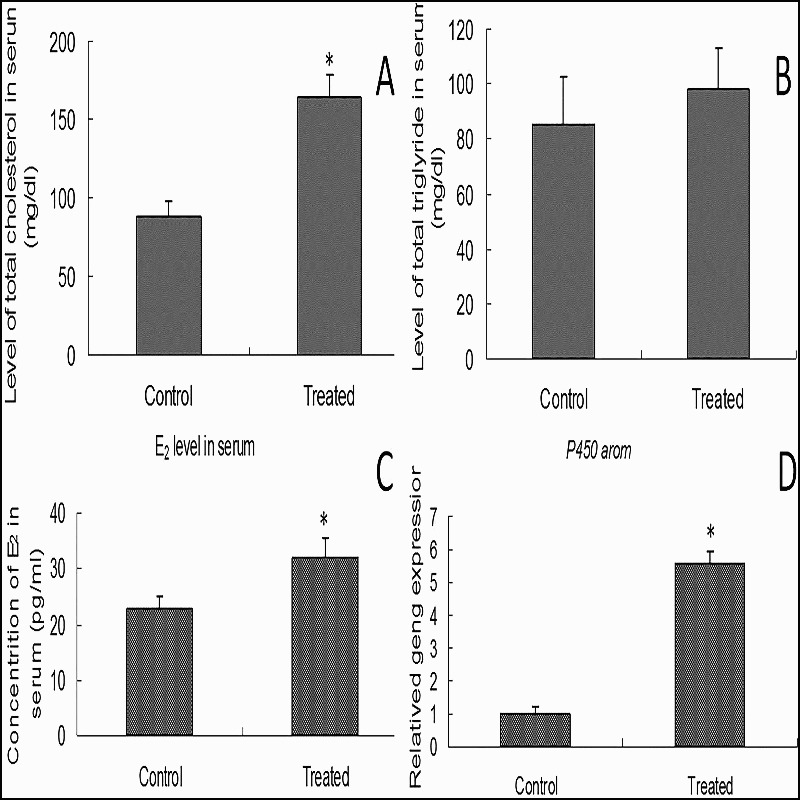
**TC, TG and E_2_ level in serum and *p450 arom* expression in cumulus.** Level of TC(A), TG(B), E_2_(C) levels in serum and the expression of *p450 arom*(D) were detected in the treated group and control group (**: *p *< .01). The data shown represent three independent experiments (mean ± SD; *: p < .05).

High-fat diet could increase the level of TC and TG in obese mouse (Wang et al. 2017). In this study, the level of TC in treated group was higher than that of the control group (163.8 ± 14.7 VS 88.5 ± 9.6; *p* < .01) (Figure 2(A)), but level of TG had no significant difference between the two group (85.0 ± 17.8 VS 98.2 ± 15.1; *p* > .05) (Figure 2(B)). E_2_ level in serum from the treated group was significantly higher than that in the control group (*p* < .05) (Figure 2(C)). Cholesterol is reverted to testosterone in follicular cells. E_2_ is mainly synthesized from testosterone by P450 arom in cumulus cells(Kato et al. 2013). The expression level of *p450 arom* in cumulus cells in the treated group was clearly higher than that in the control group (*p* < .01) (Figure 2(D)). These results demonstrated that a high-fat diet could increase TC to elevate the level of E_2_ by the catalyst of P450 arom in cumulus cells.

### Number of ovulated oocytes and parthenogenetic development

**Figure 3.**
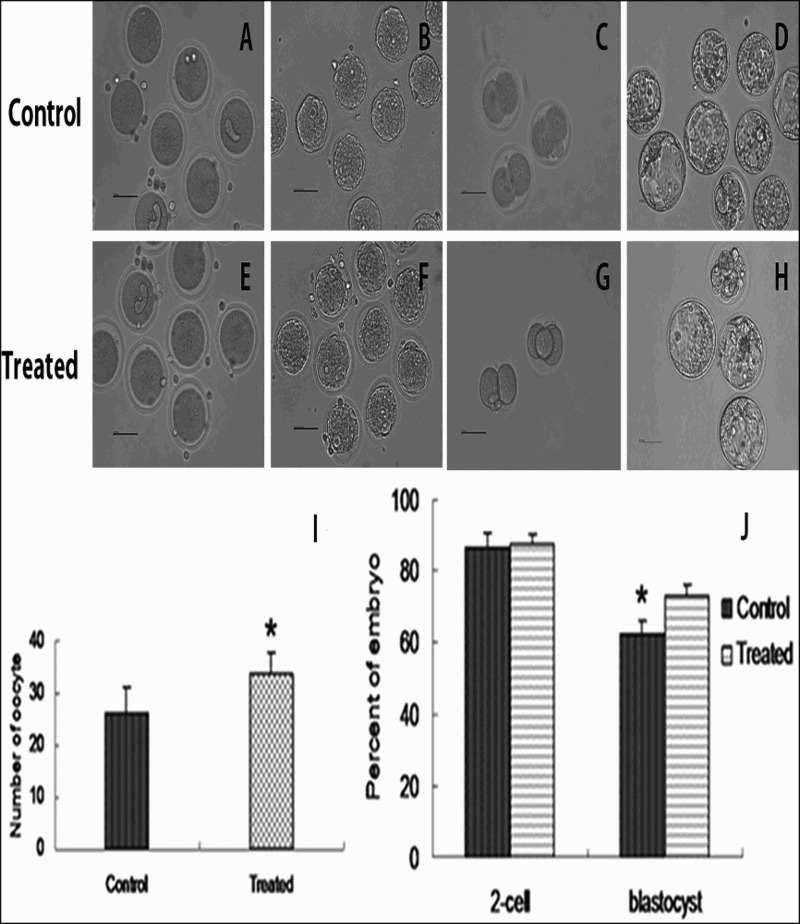
**Number of ovulated oocyte and rate of embryo development *in vitro.*** The oocytes from the control group and treated group were obtained by superovulated (A,E). The oocytes through parthenogenetic activation develop to pronuclear-stage (B,F), the pronuclear zygote (C,G) and the blastocyst (D,H) continuously. The number of ovulated oocytes was determined, for the treated and control groups (I). The percentage of embryos reaching the 2-cell and blastocyst stages, based on the number of oocytes (G). The data obtained from three independent experiments are shown (mean ± SD; *: *p *< .05).

The oocytes and embryonics from both the control group and treated group were tested by the inverted microscrope (Figure 3(A–H)). To determine the effects of a high-fat diet on ovulation and oocyte quality, we isolated oocytes from the treated and control groups for artificial parthenogenetic activation. The results showed that more oocytes were ovulated from the treated group (33.6 ± 4.8) than the control group (26.1 ± 4.0) (*p* < .05) (Figure 3(I)). There was no significant difference in the cleavage rate of parthenogenetic embryos (86.6 ± 4.3% VS 87.7 ± 3.9%, *p* > .05). However, the rate of embryos developing from the 2-cell stage to blastocysts was greater in the treated group (73.3 ± 3.1%) than the control group (62.2 ± 3.9%) (*p* < .05) (Figure 3(G)). This suggested that the quality of oocytes could be improved in vitro by a high-fat diet.

### Expression of mitochondrial and nuclear function-related genes in cumulus cells

**Figure 4.**
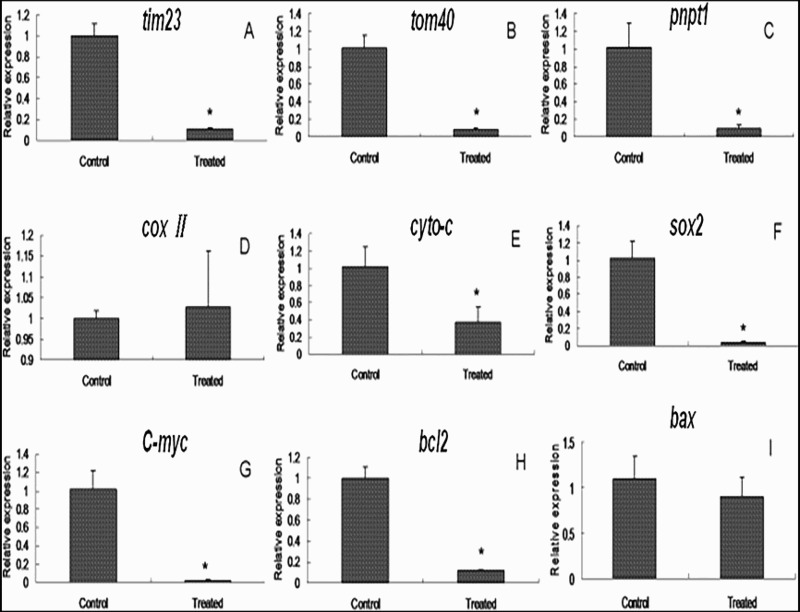
**Expression of genes in cumulus.** The expression of genes related to mitochondrial function (*tim23* (A), *tom40* (B), *pnpt1* (C), *Cox II* (D), *Cyto C* (E), and *Sox2* (F)) and nuclear function (*C-myc* (G), *bcl2* (H), and *pnpt1* (I)) in cumulus cells, from the treated and control groups. The data obtained from three independent real-time experiments are shown (mean ± SD; *: *p *< .05).

The expression of mitochondrial function-related genes as *tim23*(Translocase of inner mitochondrial membrane 23), *tom40*(translocase of the outer mitochondrial membrane 40), *pnpt1*(polyribonucleotide nucleotidyltransferase 1), *cox II* (cytochrome c oxidase II), and *cyto C* (cytochrome C) (Figure 4(A–E)) was determined by qPCR, which showed that the expression levels of *tim23*, *tom40*, *pnpt1*, and *cyto C* were significantly lower in the treated group than the control group (*p* < .05) (Figure 4(A–C,E)). For the nuclear genes, the expression levels of *Sox2*(SRY (sex determining region Y)-box 2), *c-myc* (cancer-myelocytomatosis oncogene)and *bcl2* (B cell leukemia/lymphoma 2) were significantly higher in the control group (*p *< .05) (Figure 4(F,G,H)).

### High-fat diet can decrease the mitochondrial membrane potential and increase the level of ROS

**Figure 5.**
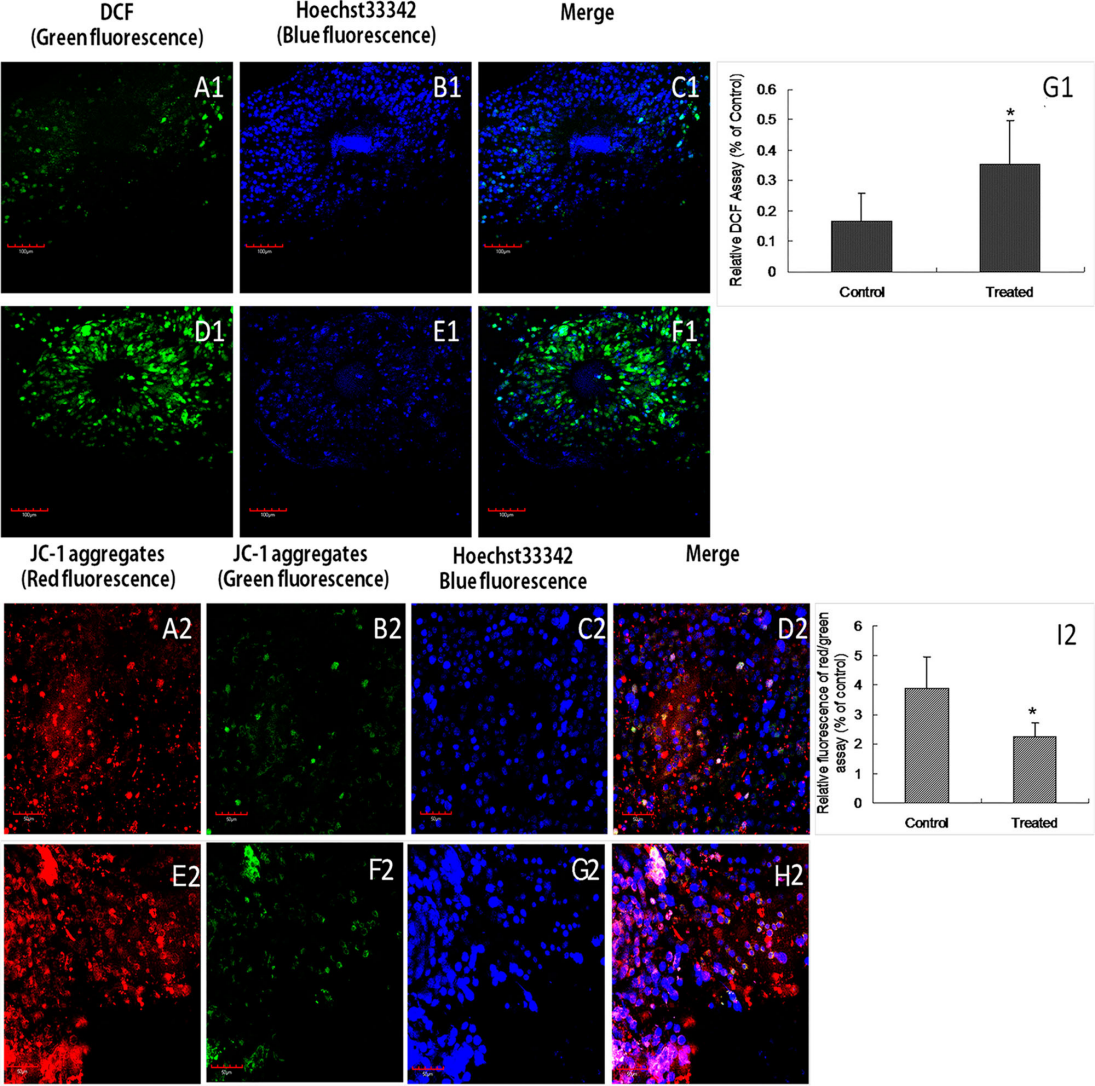
**Mitochondrial membrane potential and ROS levels of cumulus cells.** The mitochondrial membrane potential and ROS levels of cumulus cells, in control group and treated group, were obtained by confocal microscopy with the same microscope settings. To determine ROS levels, DCF staining identified the mitochondria (A1,D1), and Hoechst 33342 was used to label the nucleus (B1,E1). The fluerescences was merged (C1,F1). The relative fluorescence values based on the control were calculated for the treated and control groups (G1). Identification of the mitochondrial membrane potential. Red and green fluorescence indicate JC-1 aggregates (A2,E2) and monomers (B2,F2), respectively. Blue fluorescence is Hoechst 33342 in the nucleus (C2,G2). The three kinds of fluerescence were merged into one image. (D2,H2) The relative fluorescence intensity ratio of blue/green fluorescence based on the control is shown for the treated and control groups (I2). The data derived from three independent experiments are shown (mean ± SD; *: *p *< .05; bar: 50 µm).

The ratio of green and blue fluorescence was used to evaluate the ROS level. The ROS level was higher in the treated group compared with the control group (*p* < .05) (Figure 5 A1-G1). JC-1 was used to detect the membrane potential of mitochondria. The ratio of red and green fluorescence was used to evaluate the mitochondrial membrane potential. The mitochondrial membrane potential in the treated group was significantly lower than that in the control group (*p* < .01) (Figure 5(A2–I2)).

## Discussion

Obesity is a worldwide epidemic that could compromise human health. Obesity can impair the function of many human organs, such as the reproductive system (Kulie et al. 2011). With improvements in the living standard, humans are consuming more high-fat diets. A high-fat diet could cause obesity and other diseases (Zhou et al. 2014). Obesity could impair the function of the reproductive system, and oocytes derived from mice fed a high-fat diet had dramatically increased lipid content; thus, the quality of oocytes could be impaired (Wu et al. 2010, Luzzo et al. 2012). A high-fat diet also induced oocyte meiotic aneuploidy and fetal growth restriction/brain defects (Luzzo et al. 2012). In our primary experiment, it was intriguing to see how oocyte quality was influenced by a high-fat diet. Unexpectedly, the quality and number of oocytes were improved in mice fed a high-fat diet for 4 weeks (Figure 3), in which the possible cause is different sensitivity of the mouse strain for high-fat diet; CBA mouse is possibly easier to be damaged by high-fat diet, such as many accumulation of lipids and vacuoles in mitochondria (Wu et al. 2010). But in this study, accumulation of lipid only appeared in few cumulus, with normal structure of mitochondria but an increase of ribosome. It suggested that ICR mouse has better ability to resist damage of high-fat diet, compared with CBA mice. Therefore, oocyte quality was improved by utilization of energy from high-fat diet before appearing as lipotoxicity. Oocyte quality is an important factor in many processes, such as fertilization in vitro and in vivo, nuclear transfer and parthenogenesis. Our previous report showed that oocyte quality could be determined by artificial parthenogenetic activation, which is a simple method for detecting oocyte quality (Xu et al. 2017). Many factors are related to oocyte quality. Among these factors, the size of an oocyte is critical for developmental competence. Follicles with different sizes are responsible for oocyte development, and cumulus cells directly communicate with oocytes through gap junction. Thus, cumulus cells play an important role in oocyte developmental competence. Some studies have predicted oocyte quality from the expression profile of genes in cumulus cells, and some candidate genes have been selected (Caixeta et al. 2009). Our previous report suggested that the expression level of *Sox2* in cumulus cells was relevant to oocyte quality (Tavernier et al. 2011). However, in this study, the expression of *Sox2* did not differ between the groups. Testosterone is synthesized from cholesterol in follicular membrane cells and is converted to E_2_ in the cumulus cells by P450 arom. E_2_ is responsible for secondary sexual characteristics and influences oocyte development (Newman et al. 2008). Expression of *P450 arom* could be promoted by cholesterol in the serum (Kato et al. 2013). In this study, TC level in serum increased one fold, compared with control group, which was probably due to promoting expression of P450 arom in cumulus. It was suggested that a high-fat diet could influence the level of E_2_ in the serum by increasing the expression of *p450 arom* and level of cholesterol. It is a protective measure for cell damaged by increasing E_2_. E_2_ could protect oocyte and cumulus from clomiphene citrate-induced follicular cell apoptosis in mouse (Chaube et al. 2005). It was reported that alcohol can also significantly increase the level of free E_2_ (Schliep et al. 2015, Jerome et al. 2016). E_2_ can protect mouse mammary tissue from oxidative damage by maintaining the structure and function of proteins, lipids, and DNA (Yuan et al. 2016). In this study, the number of oocytes ovulated and oocyte quality were improved, which could be related to high levels of E_2_. Previous research has also reported that a high-fat diet can induce cell apoptosis(Moraes et al. 2009). Expression of *Bcl2* blocked apoptosis in myelodysplastic progenitors expressed as a *Bcl2* transgene (Slape et al. 2012). In this study, a high-fat diet induced low levels of expression of *Bcl2* in cumulus cells, which could lead to apoptosis in cumulus cells (Barlow et al. 2010).

Abnormal gene expression could affect mitochondrial function (Cogliati et al. 2013). In previous reports, cumulus cells and oocytes accumulated many lipid droplets in a mouse obesity model, leading to impaired oocyte quality (Wu et al. 2010, Luzzo et al. 2012). In the present study, many lipid droplets only accumulated in the cytoplasm of cumulus cells, and there were vesicles in a few mitochondria, which was in agreement with previous reports. Abnormal gene expression could be related to reduce mitochondrial membrane potential and a higher level of ROS, which was supported by a previous report (Wang et al. 2010). But the accumulation of lipid droplets did not appeared in ooplasm, which was suggested that a high-fat diet did not significantly impact oocyte. E_2_ was probably capable to resist the damage derived from the high-fat diet. Therefore, a high-fat diet induced mitochondrial dysfunction, but oocyte quality was improved. The define mechanisms need further investigated.
